# Role of DLC1 tumor suppressor gene and MYC oncogene in pathogenesis of human hepatocellular carcinoma: Potential prospects for combined targeted therapeutics

**DOI:** 10.3892/ijo.2012.1474

**Published:** 2012-05-10

**Authors:** DRAZEN B. ZIMONJIC, NICHOLAS C. POPESCU

**Affiliations:** Laboratory of Experimental Carcinogenesis, Center for Cancer Research, National Cancer Institute, National Institutes of Health, Bethesda, MD 20892, USA

**Keywords:** DLC1, tumor suppressor, MYC, oncogene, liver cancer, targeted therapy

## Abstract

Hepatocellular carcinoma (HCC) is the third leading cause of cancer death, and its incidence is increasing worldwide in an alarming manner. The development of curative therapy for advanced and metastatic HCC is a high clinical priority. The HCC genome is complex and heterogeneous; therefore, the identification of recurrent genomic and related gene alterations is critical for developing clinical applications for diagnosis, prognosis and targeted therapy of the disease. This article focuses on recent research progress and our contribution in identifying and deciphering the role of defined genetic alterations in the pathogenesis of HCC. A significant number of genes that promote or suppress HCC cell growth have been identified at the sites of genomic reorganization. Notwithstanding the accumulation of multiple genetic alterations, highly recurrent changes on a single chromosome can alter the expression of oncogenes and tumor suppressor genes (TSGs) whose deregulation may be sufficient to drive the progression of normal hepatocytes to malignancy. A distinct and highly recurrent pattern of genomic imbalances in HCC includes the loss of DNA copy number (associated with loss of heterozygosity) of TSG-containing chromosome 8p and gain of DNA copy number or regional amplification of protooncogenes on chromosome 8q. Even though 8p is relatively small, it carries an unusually large number of TSGs, while, on the other side, several oncogenes are dispersed along 8q. Compelling evidence demonstrates that DLC1, a potent TSG on 8p, and MYC oncogene on 8q play a critical role in the pathogenesis of human HCC. Direct evidence for their role in the genesis of HCC has been obtained in a mosaic mouse model. Knockdown of DLC1 helps MYC in the induction of hepatoblast transformation *in vitro*, and in the development of HCC *in vivo*. Therapeutic interventions, which would simultaneously target signaling pathways governing both DLC1 and MYC functions in hepatocarcinogenesis, could result in progress in the treatment of liver cancer.

## Contents

1. Introduction
2. Hepatocellular carcinoma, a common cancer worldwide
3. Genomic deletion and tumor suppressor genes
4. Genomic overrepresentation and oncogenes
5. Genomic and related cancer gene alterations on chromosome 8 in hepatocellular carcinoma
6. DLC1 a potent tumor suppressor gene in hepatocellular carcinoma
7. MYC, a major oncogene in cancer
8. Critical role of MYC in the pathogenesis of human and mouse hepatocellular carcinoma
9. Modulation of DLC1 and MYC in hepatocellular carcinoma: prospects for combined pharmacologic interventions

## 1. Introduction

Genomic instability and several other hallmarks of cancer underline the complexity of neoplastic disease (1). During the process of cancer development, the expression output of a variety of genes can be modulated by mutations, amplifications, deletions, and chromosome translocations; these changes accumulate in time and materialize in the appearance of an incipient abnormal cell with the capacity to perpetually proliferate (2). Advances in cytogenetics and molecular biology have led to the establishment of the genetic basis of neoplasia and to the recognition that chromosomal alterations affect genes critical in the pathogenesis of human cancer. The detection of recurrent chromosomal alterations has greatly facilitated the identification of genes important in oncogenesis and provided the basis for development of highly efficient cancer therapeutic agents. The best known examples are the development of Imatinib and Herceptin therapies for chronic myelocytic leukemia (CML) and breast cancer, respectively. Imatinib was consequential to identification of a bcr-abl fusion protein resulting from specific chromosome translocation between chromosomes 9 and 22 in CML, whereas development of Herceptin followed the detection of recurrent HER/Neu (ErbB2) amplification on abnormal chromosomes in breast cancer cells (3,4). Both examples underline the importance of searching for gene alterations at the sites of recurrent chromosome abnormalities.

Epigenetic changes are equally important to the process of cancer development. In many types of cancer, tumor suppressor genes (TSGs) are frequently downregulated or silenced by promoter hypermethylation or histone deacetylation (5). In the past few years a new trend emerged in cancer therapy with focus on functional alterations mediated by epigenetic mechanisms. Therapeutic approaches that reverse the adverse effect of epigenetic modifications have already been developed, such as Vidaza and Decitabine, both potent DNA methyltransferase inhibitors, or Vorinostat, a histone deacetylase inhibitor. These therapeutics are applied either alone or in combination (6,7).

## 2. Hepatocellular carcinoma, a common cancer worldwide

Human hepatocellular carcinoma (HCC) is one of the most common cancers worldwide, accounting for 90% of all liver neoplasias. It is the third leading cause of cancer death, and its incidence is increasing in several countries, including the United States (8,9). Infection with hepatitis B (HBV) and C viruses (HCV), consumption of aflatoxin-contaminated foods and/or alcohol, and exposure to other chemical carcinogens have been implicated in the etiology of HCC (8). In the multistep process of HCC development, both the chemical carcinogens and the oncogenic viruses can cause DNA damage, which is manifested at the chromosome level as deletions, duplications, and translocations, frequently affecting the structure and expression of cancer-related genes. With the use of a combined approach based on integration of molecular cytogenetics and molecular biology, our investigations focused on identification and characterization of recurrent chromosome alterations that have led to the discovery of new cancer-related genes as well as to the detection of alterations in known ones. Our group and many others searched for recurrent and specific genomic and gene alterations in HCC and examined both the karyotypes and the quantitative profiles of genomic imbalances and gene expression of cell lines or primary tumors. A recent article underlining the role of TSGs as metabolic regulators stated that cancer therapy is increasingly shifting toward therapeutics based on genetic alterations displayed by cancer cells (10).

Here we summarize the progress and our contribution toward uncovering genomic and related gene alterations as targets for therapeutic interventions, and focus on the role of two genes, MYC oncogene and deleted in liver cancer 1 (DLC1) gene, in hepatocarcinogenesis.

In our early studies, we identified novel nonrandom chromosome rearrangements and a distinct pattern of multiple recurrent DNA copy-number gains and losses, some of which mimicked alterations frequently seen in other neoplasias and those potentially specific for HCC (11,12). Such analyses significantly expanded the map of genomic changes in HCC and led to the identification of genes that may play an important role in the pathogenesis of HCC. We examined the karyotypes of a number of HCC cell lines and, by using approaches such as spectral karyotyping (SKY), comparative genomic hybridization (CGH), and array-based CGH (aCGH) analyses, we were able to identify nonrandom chromosomal alterations and recurrent breakpoints in balanced and unbalanced rearrangements, as well as to precisely map recurrent genomic amplifications and deletions (11,12). It became apparent that not all the genomic sites were equally affected by gain or loss of DNA and that the regions known as fragile sites (FSs) were most frequently involved. In the past several years, molecular and cytogenetic evidence for cancer-specific translocations, amplification of oncogenes, deletion of TSGs, and viral integration at FSs firmly implicated these regions of fragility and recombination in cancer development (13). A half of all microRNA genes, whose transcripts (mRNAs), may act as either oncogenes or TSGs, and whose expression is deregulated by amplification, deletion, mutation, and epigenetic modifications in a variety of cancers, are located within or near FSs (14).

## 3. Genomic deletion and tumor suppressor genes

Molecular cytogenetic analysis of HCC cell lines revealed nonrandom deletions and unbalanced translocations of chromosomes 1 and 3, with the breakpoints clustered at regions 1p36 and 3p14-21, close to the loci of p73 and FHIT TSGs, respectively (11). This report was the first to describe unbalanced translocations of chromosomes 1 and 3 with the breakpoints nonrandomly involving these loci in HCC. Unlike in balanced abnormalities, unbalanced alterations may result in loss of genes. Cytogenetic abnormalities of chromosome 1 are common in HCC, and several lines of evidence implicate alterations of specific sites on its short arm in the pathogenesis of HCC (15,16). Region 1p36 is a region of fragility and recombination and is suspected to harbor multiple TSGs. Because several oncogenes and at least two cell senescence genes are located on chromosome 1, it has been postulated that structural alterations and the consequential imbalance of chromosome 1 may be important for gene dosage in certain types of cancer, including HCC (12). Recently, using an approach based on integrated genomic data of DNA copy number and gene expression profiles, researchers identified several potential driver genes on chromosome 1 in HCC (17).

In addition to 1p36, another site of recurrent translocation involves the region 3p14-21 where the FHIT gene is located. The FHIT gene is expressed in normal hepatocytes but is either abnormally expressed or inactivated in HCC cells. We detected recurrent chromosome 3p rearrangements, a decrease or absence of FHIT mRNA expression, intragenic deletions, and an absence of protein expression. These observations strongly suggested that FHIT alterations might be pathologically relevant to HCC. Chemical carcinogens or HBV integration at FRA3B may initiate a background of genetic instability early in the process of hepatocarcinogenesis (18). In HCC cell lines, we also identified recurrent DNA loss on the short arm of chromosome 3 at sites other than 3p21 (12).

Among other candidate TSGs, located at regions of deletion in 3p in various cancers, is the TMEM7 gene, which encodes a transmembrane protein (19). This gene is expressed specifically in the liver, and its protein shares substantial sequence homology with human and mouse 28-kDa interferon-α (IFN-α) response modifier protein. We investigated the role of TMEM7 in the development of human HCC and demonstrated that, in the absence of genomic deletion and mutation, the downregulation or silencing of the gene is due to aberrant DNA hypermethylation and histone deacetylation. Ectopic expression of TMEM7 inhibited HCC cell proliferation, colony formation, and cell migration *in vitro*, and reduced tumor formation in nude mice. Treatment of two highly invasive HCC cell lines with IFN-α significantly increased TMEM7 expression and inhibited cell migration. These observations implicate loss of TMEM7 expression in hepatocarcinogenesis, and suggest that modification of TMEM7 expression by IFN-α may have potential therapeutic relevance in a subset of HCC (20).

During the neoplastic process, certain tumor cells acquire resistance to the antiproliferative signaling of TGFβ. We examined a human HCC cell line sensitive to TGFβ1 (Hep3B-TS), and its derivative, Hep3B-TR, rendered resistant to TGFβ1 by stepwise exposure to the agent (21). SKY and aCGH analysis showed that the TGFβ resistance resulted from a loss of TGFβ receptor II (TGFβRII) gene, which occurred when the only remaining apparently normal chromosome 3 in Hep3B-TR cells, underwent microdeletion encompassing the site of the gene. A comparative differential gene expression analysis of the above-mentioned cell lines, using an oligonucleotide microarray, identified six genes in Hep3BTR cells that are downstream targets of the TNF gene, suggesting that loss of TGFβRII triggered the activation of the tumor necrosis factor network known to be regulated by the TGFβ1 pathway. Functionally, loss of TGFβRII in cells resistant to TGFβ1 significantly enhanced cell migration and anchorage-independent growth *in vitro*, and also increased *in vivo* tumorigenicity compared with parental sensitive cells (21).

Deletion and loss of heterozygosity (LOH) at specific regions of the long arm of chromosome 16 are common in several forms of cancer, including HCC (8). We have focused on region 16q24 that harbors tumor suppressor gene WWOX, which spans FRA16D, the second most common FS (22). The status of WWOX genomic DNA, as well as of the transcribed RNA and translated protein, was examined in HCC cell lines, and recurrent alterations of the gene have been identified. Loss of DNA copy number confined to band 16q23 was detected by CGH in several cell lines (12). Although homozygous deletions of WWOX were not detected, WWOX mRNA was either absent or reduced in 60% of the cell lines examined. The detection of aberrant RT-PCR products of WWOX transcripts, with deletion of exons 6 to 8, correlated significantly with altered WWOX expression. All of the cell lines showing downregulation of WWOX mRNA, also had a reduced or undetectable level of WWOX protein. Furthermore, in a majority of the HCC cell lines, the overall amount of WWOX protein was markedly reduced or undetectable in comparison with that of a normal liver. These results show that WWOX is frequently altered in HCC, and therefore implicate it in hepatocarcinogenesis (23). Because carcinogenic agents preferentially target common FSs, it is possible that breakage of WWOX locus at FRA16D and of FHIT gene at FRA3B occurs concomitantly in certain HCCs.

## 4. Genomic overrepresentation and oncogenes

In our CGH analysis of HCC cell lines, several regions of recurrent DNA copy-number gains have been identified (12). The detection of two regions of DNA overrepresentation on 11q13 and 5q31, both overlaping with the locations of common FSs, led us to take a closer look at two genes, EMS1 and SMAD5, that reside at/or close to those chromosomal spots. Region 11q13 harbors EMS1 oncogene and FRA11H. We found that EMS1 is amplified in primary HCC and overexpressed in HCC cell lines in the absence of gene amplification. This oncogene encodes cortactin, a cortical actin-associated protein that is a substrate for the tyrosine kinase Src and contributes to reorganization of the actin cytoskeleton. Alterations of EMS1 that lead to the overproduction of cortactin may thus be important in the development of HCC. EMS1 amplification and overexpression are indicative of an unfavorable prognosis in several cancers and may have similar prognostic implications in liver cancer (24). The other earlier-mentioned minimal region of DNA copy-number gain in HCC, identified at chromosome 5q31, overlaps with the location of FRA5C and with the locus of the SMAD5 gene (25). Deletions at this location, unbalanced translocations with breakpoints near the SMAD5 locus, recurrent formation of isochromosome 5q resulting in selective loss of 5p and gain of 5q, and intrachromosomal amplification of SMAD5 at FRA5C have been detected; all point to this locus as relevant in the development of HCC. High-level amplification of SMAD5 at FRA5C is one of the few examples of gene amplification at a common FS mediated by breakage-fusion-bridge cycles (13). These observations show that SMAD5 undergoes gain in copy number, high-level amplification, and overexpression rather than loss of expression, suggesting that it does not function as a TSG in HCC (25).

## 5. Genomic and related cancer gene alterations on chromosome 8 in hepatocellular carcinoma

The genes thus far identified in our studies, which promote or suppress tumor cell growth, most likely represent only a fraction of possible future therapeutic targets. In spite of wide heterogeneity of genomic alterations in HCC, certain chromosomes or chromosomal sites are more commonly deleted or amplified, which results in deregulation of critical genes that, ultimately, may trigger transformation of normal hepatocytes into malignant phenotype. The best example of the above, perhaps, are genomic alterations of chromosome 8 in HCC. This chromosome shows a distinctive pattern of both recurrent of DNA copy number loss on 8p, and gain on 8q (12). Due to the high frequency of large genomic deletion and LOH in HCC and several other cancers, chromosome 8p has been long suspected to carry TSGs. Moreover, LOH on chromosome 8p is among the most common alterations in HCC and is associated with liver dysplastic nodules and primary and metastatic HCC (26–32). Chromosome 8p contains two FSs and is rich in candidate and validated TSGs, some of which have been implicated in the pathogenesis of HCC (33,34). Region 8p22 alone harbors six TSGs (Table I). Among the candidate and bona fide TSGs implicated in HCC are PDGFRI, DLC1, LFIRE/HFREP-1, SFRP1, MSRA, and newly identified SCARA5 gene (35–41).

Liver fibrinogen-related gene-1, LFIRE-1/HFREP-1, specifically expressed in normal human liver tissue, is recurrently downregulated or inactivated and has an antiproliferative effect on HCC cells both *in vitro* and *in vivo*(37).

SFRP1, a secreted Wnt antagonist, and SCARA5, a plasma membrane protein, which activate the focal adhesion kinase (FAK) signaling pathway and inhibit the tyrosine phosphorylation cascade of the FAK-Src-Cas pathway, are strong candidate tumor suppressor genes due to their antiproliferative effect on HCC cells (39,41). Since its isolation, SFRP1 gene was predicted to be a TSG because of its location at 8p11-12, a site of LOH in HCC (42). However, in many types of cancer, including HCC, SFRP1 has been found to be silenced primarily by hypermethylation, and not by genomic deletion (39,43). In a few instances, restoration of SFRP1 expression resulted in reversal of the malignant phenotype (39,43). However, one study reports that SFRP1 is upregulated in metastatic renal cell carcinoma and that it contributes to the invasiveness of the tumor cells (44). The same researchers and others had reported that SFRP1 is frequently downregulated in primary renal cell carcinoma (45). The probability that SFRP1 downregulation contributes to hepatocarcinogenesis (39) is rather high, given the role and importance of Wnt signaling in the pathogenesis of HCC (46,47). A study demonstrating the suppressive effect of SCARA5 on tumor cell proliferation *in vitro* and on metastasis *in vivo*(41), indicates that this gene also may play an important role in initiation and development of HCC.

Other characterized TSGs such as N33, TRAIL-R1, TRAIL-R2, LZTS2, hBD-1, DBC2, DOK, and NRG1, are also located on 8p, but none of them was examined in HCC. Three new genes located on 8p, SORBS3, SHRBS3 and PROSC, have been characterized quite recently and their function will be discussed later in the text, within the contest of TSG role in HCC. A number of oncogenes are located on 8q, but only MYC is closely associated with the development of HCC (Table I). Among all cancer-related genes on chromosome 8, DLC1 and MYC play a major role in hepatocarcinogenesis, and evidence for their involvement in the development of HCC is presented next.

## 6. DLC1 a potent tumor suppressor gene in hepatocellular carcinoma

The DLC1 gene was isolated from a chromosomal fragment deleted in a human HCC, and was suspected to be a TSG (36). Since its isolation, the interest in this gene has grown considerably worldwide. Progress in understanding of its tumor-suppressive function in multiple cancers, particularly in HCC, will be discussed here. Two closely related genes, DLC2 and DLC3, also were isolated and were found to be frequently deregulated and to elicit oncosuppressive effects in HCC (48,49). Until now, three DLC1 isoforms, called variants 1, 2, and 3 (or α, β and γ), were isolated (50). Most recently, a new isoform 4 (DLC1-i4) was identified and shown to suppress tumor cell growth, with its functional promoter regulated by p53 (51).

In recent years, DLC1 has emerged as a major tumor suppressor gene and a strong candidate metastasis suppressor gene in HCC and other cancers (50,52–55). DLC1 is also a contributing factor in processes affecting normal functions, or disorders unrelated to cancer. DLC1 is vital for normal development because null mutations in the gene result in embryonic lethality in mice and flies. It is also involved in insulin signaling and the pathogenesis of coronary spastic angina and is among four genes that negatively regulate periodontal ligament cell proliferation (56–59).

The DLC1 gene is located on chromosome 8p22, a region (Table I) of recurrent LOH in HCC (36). Although region 8p22 does not correspond to an FS, its propensity for deletion is similar to that of the most unstable and vulnerable FSs. Downregulation or inactivation of TSGs has profound consequences in the genesis and progression of cancer, and DLC1 is one of the most frequently deregulated genes in the cancer genome. Downregulation or inactivation of the DLC1 gene in many forms of cancer, is commonly mediated at the genomic level by heterozygous and homozygous deletion, and at the transcriptional level by aberrant promoter methylation or histone deacetylation (50). Initially, we detected lack of DLC1 expression in over 40% of human primary HCC and in 90% of HCC cell lines (36). These results were independently confirmed, as 40% of HCC samples had no detectable DLC1 expression (60). More recently, using representational oligonucleotide microarray, heterozygous deletions of DLC1 were found in 59 of 86 primary HCCs. Furthermore, deletions of DLC1 occurred in other cancers more frequently than of some other well-known TSGs (53). In contrast with the early observation that mutations in the coding region of DLC1 are rare in HCC, recent genome-wide sequencing analyses of several cancers have identified missense mutation of DLC1 (61–64). In an early study of HCC cell lines, we identified a number of single nucleotide polymorphisms in the DLC1 genomic DNA sequence, which may be associated with an increased risk for development of HCC (61). Only recently, a comprehensive genotyping analysis of a large number of cases in the Chinese population provided convincing evidence for an association between DLC1 polymorphism and susceptibility to HCC. Furthermore, evidence shows that the DLC1 polymorphism and risk for HCC development are consistent with its biological function and strongly suggest that the DLC1 polymorphism may directly confer susceptibility to HCC (65).

Promoter hypermethylation appears to be associated with human cancer at least as frequently as is disruption of TSGs by mutation or deletion. TSGs that are downregulated or silenced by promoter hypermethylation are often located in genomic regions that are frequently deleted in cancer (13,66). Despite a high rate of deletion, DLC1 is predominantly silenced or down-regulated by epigenetic mechanisms, frequently by promoter methylation (50,67,68). An expression profile of the three DLC1 isoforms - variants 1, 2, and 3 - in normal and malignant hepatic tissue, showed that only variant 1 was silenced by promoter methylation; variants 1 and 2 (but not variant 3) localize at focal adhesions and suppress stress fiber formation and *in vitro* HCC cell proliferation (69). DLC1 variant 1 is not the only one silenced by promoter methylation. As recently demonstrated with the newly identified isoform 4, DLC-i4 is expressed in normal tissues and normal immortalized cell lines but is significantly downregulated in a high number of nasopharyngeal, esophageal, gastric, breast, colorectal, cervical, lung carcinoma cell lines as well as in primary tumors. Methylation of DLC1-i4 was detected in 38% of primary HCCs (51).

In addition to deletion, mutation, and promoter methylation, DLC1 can be downregulated or silenced by other mechanisms. DLC1 did not escape the action of certain miRNAs that target TSG expression (70). A new study of significant interest and with implications for the role of HCV in chronic liver disease and HCC, has demonstrated that efficient HCV replication requires miR-141-mediated suppression of DLC1. In primary human hepatocytes, HCV infection stimulated cell proliferation that was reversed by transduction of DLC1 (71).

**Figure 1:**
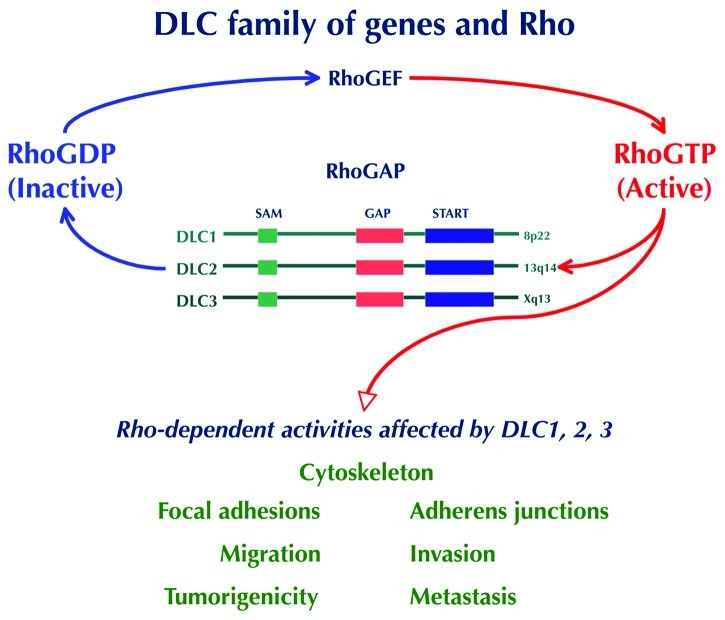
Small RhoGTPases alternate between their active (RhoGTP) and inactive (RhoGDP) forms. Activation is controlled by guanine nucleotide exchange factors (GEFs), whereas deactivation is mediated by GTPase activating proteins (GAPs) Rho effector proteins, which include DLC protein family, play an important role in processes that affect cell morphology, motility and tumorigenesis.

DLC1 encodes a RhoGAP protein that catalyzes the conversion of active GTP-bound Rho GTPase (Rho) to the inactive GDP-bound form (Fig. 1). DLC1-mediated inhibitory effect of cell growth and tumorigenicity is primarily due to its RhoGAP’s ability to inactivate the Rho proteins - RhoA, RhoB, and RhoC, and to some degree Cdc42, but not Rac - although RhoGAP-independent mechanisms also contribute to its antioncogenic activity (72–80). Rho GTPase activity is frequently deregulated in human cancers, and experimental evidence implicated Rho GTPase signaling in promoting growth and invasiveness through morphologic and functional cellular alterations such as cell shape, actin cytoskeleton remodeling and focal adhesion organization, as well as cell migration and invasion (50,81,82). Because the activity of Rho GTPases is elevated in many human cancers, the ability of RhoGAPs to downregulate Rho proteins may attenuate or inhibit tumorigenic and/or metastatic process (83–85). The importance of altered Rho GTPase signaling in the genesis and progression of HCC has been previously covered in detail (85). Special attention was given to DLC1, whose loss of function was considered a driving event in the promotion and progression of HCC. DLC1 also was recognized as the best example of a RhoGAP alteration in HCC (85). Most recently, in an integrative genomic and transcriptomic profiling of HCC from 76 patients with HBV infection designed to identify survival-related driver genes, DLC1 and five other genes on 8p were found deleted in patients with poor outcome. Interestingly, three of these genes, SORBS3, SHRBS3 and PROSC act as TSGs and suppress HCC *in vitro* and *in vivo*(86).

Indeed, highly recurrent alterations of DLC1 have been found in solid tumors and hematologic malignancies, and a meta-analysis of microarray experiments lists DLC1 as the fifth of the top-50 genes implicated in multiple cancers (87).

DLC1-transduced HCC cells display a diffuse cytoplasmic localization of the protein (72,88), whereas in other cells DLC1 protein co-localizes with the tips of actin stress fibers and in focal adhesions, which is critical for cell migration (72,75,88–90). Evidence that DLC1 functions as a TSG in HCC comes from experiments in which DLC1 cDNA was ectopically expressed in cells with disabled endogenous gene expression. In several independent studies, restoration of DLC1 expression invariably resulted in inhibition of cell proliferation, colony formation, and cell migration *in vitro*, and it reduced the development of tumors after xenografting of HCC cells in athymic nude mice (60,72,74,88). Two well-characterized cell lines, derived from patients with aggressive HCC, and negative for endogenous DLC1 activity, were used to test the antiproliferative effect of the DLC1 ectopic expression. Restoration of DLC1 expression resulted in inhibition of *in vitro* cell proliferation and *in vivo* tumorigenicity, as well as in induction of apoptosis associated with cleavage of caspase 3 and a reduced level of the antiapoptotic Bcl-2 (88). Subsequent studies with prostate cancer showed that tumor cells acquire sensitivity to DLC1-induced apoptosis after treatment with HA14-1, a Bcl-2 inhibitor (76). It has been demonstrated that subcellular locations of DLC1 protein determines different manifestations of its antioncogenic action; in cytoplasm DLC1 functions as an inhibitor of tumor cell proliferation and migration, whereas in the nucleus it acts as an inducer of apoptosis (91).

In the past few years, evidence supporting the metastasis suppressor function of DLC1 has been generated with breast cancer, and HCC cells (77,92,93). The role of DLC1 in metastatic process will be covered in a separate article.

A yeast two-hybrid screening was invaluable to further dissect complex molecular machinery underlying DLC1 functions. While the multidomain structure of DLC1 merely indicated its capacity to interact with other molecules, the yeast two-hybrid analysis identified several potential binding partners of DLC1. Those binding partners were later confirmed in human cells, allowing the examination of consequences of their interactions with DLC1. Such was the case with the tensin family of focal adhesion proteins, which serve as a link between the actin cytoskeleton and the cytoplasmic tails of integrins, whose relationship with DLC1 has been thoroughly investigated in different types of tumor cells (94–100). In human lung cancer cells, it was demonstrated that DLC1 binds to SH2 and PTB domains of the tensin proteins and, most importantly, that cooperation between DLC1 RhoGAP action and its tensin-binding activity is required for suppression of cancer cell migration, although the two functions are not interdependent (75).

Another group reported that tensin 2 and DLC1 form a complex, which binds to caveolin-1 (Cav-1), a structural component of caveolae. Such binding brings DLC1 in close proximity to locally enriched RhoGTPases and facilitates the inactivation of Rho proteins through the RhoGAP activity of DLC1 and with repercussions at the level of cytoskeleton. Interaction of this kind would constitute a potential new mechanism of DLC1 action in hepatocytes, through cytoskeleton reorganization (94). Cav-1 functions as a TSG, and recently Cav-1 interaction with the START domain of DLC1 was identified and shown to contribute to the tumor suppressor activity of DLC1 (101). We also detected DLC1 interactions with p120RasGAP, α-catenin, and S100A10 proteins in human cells, and assessed their influence on DLC1’s tumor suppressor function (102–104). The interaction of DLC1 with p120RasGAP protein, which is implicated in cross-talk between Ras and Rho, not only provided relevant data for the negative modulation of DLC1 tumor suppressor activity but may, also, lead to potential clinical interventions (102). The interaction was mapped to the RhoGAP catalytic domain of DLC1 and the SH3 domain of p120RasGAP, and resulted in a dramatic reduction of DLC1’s RhoGAP activity *in vitro*. Moreover, overexpression of p120RasGAP in colon carcinoma cells that harbored mutant Ras and thus were resistant to the negative regulation of Ras by p120RasGAP, increased the level of endogenous active Rho in a DLC1-dependent manner and antagonized the growth-suppressive effects of DLC1. These data show that p120RasGAP can promote the growth of cells containing mutant Ras, and are consistent with evidence suggesting that it might represent a valid therapeutic target for tumors carrying a mutant Ras, which account for approximately 30% of all tumors, and for which an efficient cancer treatment to block Ras function did not translate in clinical interventions (55). Thus, p120RasGAP-targeting therapy might be effective also in a subset of HCC with mutated Ras (102).

In contrast with the negative effect of interaction with p120RasGAP on DLC1 tumor suppressor function, preliminary evidence indicates that DLC1 interaction with α-catenin enhanced DLC1 anti-oncogenic activity (103).

We reported DLC1 interaction with S100A10, an inflammatory protein and a key cell surface receptor for plasminogen that regulates pericellular proteolysis and tumor cell invasion. DLC1 and annexin 2 share the same binding site at the C-terminus of S110A10. DLC1 binding to S100A10 caused an dose-dependent decrease in steady-state S100A10 expression by displacing it from annexin 2 and making it accessible to ubiquitin-dependent degradation. This process attenuated plasminogen activation and was accompanied by inhibition of *in vitro* cell migration, invasion, colony formation, and anchorage-independent growth of aggressive lung cancer cells. DLC1 binding to S100A10 did not affect DLC1’s RhoGAP activity. Thus, this study unraveled a novel GAP-independent mechanism that contributes to the tumor suppressor activity of DLC1 (104).

Although transcriptional regulation of DLC1 through genetic and epigenetic alterations was discussed in detail, only recently have posttranslational modifications been reported, mediated by phosphorylation of a serine-rich domain, an unstructured region of DLC1 gene, and shown to negatively regulate the anti-oncogenic function of DLC1 (93,105). Unstructured domains are preferred sites for posttranslational modifications, including phosphorylation, that target multiple sites in the DLC family proteins (50,106). In an earlier study, Ser322 of rat DLC1 was found to be phosphorylated in rat adipocytes after insulin treatment, and posttranslationally, rat DLC1 has been shown to be phosphorylated by Akt kinase (57). This finding set the stage for investigations of the role of Akt phosphorylation of DLC1 on suppression of tumorigenicity and metastasis in HCC (93).

The 14-3-3 protein is known to function as a phosphorylation-dependent adaptor protein that facilitates interactions with other proteins (107). It is interesting that activated protein kinase D (PKD) phosphorylates DLC1 and in turn facilitates interaction with 14-3-3 protein isoforms. Binding of 14-3-3 protein inhibited DLC1 RhoGAP activity and prevented nucleocytoplasmatic shuttling and, thus, may impede the DLC1 apoptotic function (91,108). As a follow-up to this study, the same group provided evidence showing that PKD phosphorylation of Ser 807 within the GAP domain negatively regulates DLC1 function (105).

In summary, the role of DLC1 is rather complex, and, as recognized by others, intricacies of the DLC1 tumor suppressor function and its modulation by multiple and elaborate interactions should stimulate interest and new research in this TSG (109). DLC1 meets the criteria for a gatekeeper gene because of its inhibitory effect on tumor cell growth and cell death (110). Given the unusually high number of TSGs on the short arm of chromosome 8, the inhibitory effect of DLC1 on tumor cell growth may compensate for loss of function of other TSGs. Evidence presented here confirms DLC1 as an important gene in the development and progression of HCC.

## 7. MYC, a major oncogene in cancer

MYC protooncogene, the human cellular homologue of the avian myelocytic leukemia virus, a retroviral oncogene, is a transcription factor that regulates cell proliferation, controls the expression of many genes, and is implicated in the pathogenesis of a plethora of human solid tumors, leukemias and lymphomas, as well as in animal tumors. MYC is a powerful cancer-promoting gene but, paradoxically, also shows oncosuppressive activity by inducing apoptosis and cell senescence. Since its isolation 27 years ago, MYC has commanded interest worldwide, and nearly 20,000 studies on virtually every form of cancer have been published. A recent comprehensive article reviewed the progress in understanding MYC function; the mechanisms contributing to the induction of cell transformation, apoptosis, gene deregulation, and control of expression; and the identification of MYC target genes (111). Therefore, we will briefly outline the general aspects of MYC’s role in cancer development and present our encounter with this gene in various cancers, particularly in HCC.

Abnormal MYC expression is a common denominator in cancer, and its activation is mediated by insertional mutagenesis, chromosomal translocations and gene amplification, but not by mutations in the coding sequence (111). Insertional mutagenesis, the process of insertion of new sequence(s) into a normal gene, frequently occurs during integration of retroviral proviruses into the host genome, resulting in chimeric sequences of both viral and cellular origin. Retroviruses are obligate mutagens because their life cycle require integration into the host’s chromosomal DNA (up to 8% of human genome is of retroviral origin), an event that, in addition to structural alterations at the site of impact, also may activate adjacent cellular oncogenes (111–114). MYC activation by retroviral insertion was the first demonstration of such phenomena (115). Unlike retroviruses, the integration of certain DNA viruses into chromosomal DNA, being not required for viral persistence, was long considered rather an exception, than a rule. Abundant evidence for integration of several oncogenic DNA viruses into the cellular genome, obtained from a variety of human cancers, has challenged this view. As already mentioned, their integration, apparently, is not a random event, because they preferentially target FSs, which frequently encompass the location of growth-regulatory genes (116). Integration of oncogenic DNA viruses, such as of human papillomavirus (HPV) in most invasive genital cancers, Epstein-Barr virus (EBV) in infected lymphoblastoid cells or Burkitt’s lymphoma (BL), and adeno-associated virus (AAV) targeting MYC locus, have been well documented (13). The most recent evidence of specific viral integration at FS was the localization of simian virus 40 (SV40) in SV40-immortalized cells, at region 1q21.1, which corresponds with the location of a common FS (FRA1F). SV40’s insertion in the cell’s genome caused deregulation of adjecent genes involved in senescence and apoptosis and, thus, played a critical role in cellular immortalization (117).

The specificity of viral integration is fundamental to determining the biological significance of this phenomenon in various pathologic conditions, including cancer. The introduction of viral genetic material and its interaction with the cellular genome may contribute to the induction of cell transformation, the maintenance of the transformed phenotype, or tumor progression (113,114,118). Undoubtedly, the most conclusive evidence for the effect of viral integration at FSs in cancer development was provided in a study of young patients with X-linked severe combined immunodeficiency, when three of ten children developed monoclonal acute lymphoblastic leukemia- like lymphoproliferation after gene therapy that used murine leukemia virus-derived vector as a gene delivery vehicle. It has been unequivocally demonstrated that in two of those patients (and probably in all three) the activating vector integrated within FRA11E region, near the transcription start site of the LMO2 oncogene (119), thus causing high levels of transcription and, consequently, of *LMO2* protein.

Chromosomal translocations, isochromosomes, double minute chromosomes (DM), and abnormally banded or homogeneously stained regions (HSRs) are also responsible for MYC deregulation in a variety of cancers. Chromosomal translocations are common in leukemias and lymphomas, and the best known examples are reciprocal translocations in BL involving the MYC locus at FRA8C to any of the three immunoglobulin genes on chromosome 2, 14, or 22, resulting in MYC gene deregulation. Juxtaposition of MYC with immunoglobulin loci was causally related to development of lymphomas (120). In addition to the reciprocal translocation t(8;14), in two well-characterized BL cell lines we identified two more complex translocations, t(8;14;18) and t(7;8;14). These novel rearrangements, a manifestation of genomic instability, resulted in transposition of MYC sequences in a new genomic context and, together with duplication of chromosome 1q, which is the second most common alteration in BL, may have contributed to the acquisition of an invasive tumor phenotype (121).

**Figure 2:**
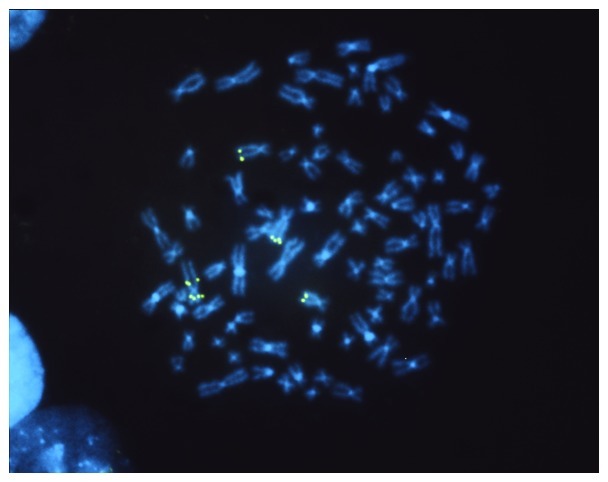
Metaphase from HCC cell line 7703K, with an abnormal chromosomal complement, was hybridized *in situ* with a genomic MYC probe. Multiple fluorescent signals for MYC gene, indicative of gene amplification, are located at normal and abnormal chromosome 8.

An example of an increased copy number of MYC on normal and rearranged chromosome 8 in HCC is shown in Fig. 2.

Whereas abnormal isochromosomes cause a modest increase in MYC copy number, DM and HSR may generate high-level amplification, commonly found in human solid tumors (122,123). HSR may contain two or more amplified genes, frequently oncogenes. In breast cancer cells, for example, using DNA fibers hybridized with genomic probes, we detected high-level amplification not only of MYC but of ERBB2, as well (124). Nonrandom chromosome alterations resulting in amplification of MYC gene also have been identified in normal human mammary cells neoplastically transformed by the controlled introduction of SV40LT, hTERT, and H-RAS genes, thus implicating MYC in the early stages of neoplastic development (125).

## 8. Critical role of MYC in the pathogenesis of human and mouse hepatocellular carcinoma

Evidence from genetic analyses of human early liver lesions, primary and metastatic liver tumors, tumor-derived cell lines, and from different rodent models, unequivocally demonstrated that MYC deregulation is a critical alteration in the initiation and progression of HCC. A recent gene expression profiling of cirrhotic and dysplastic nodules and early HCC, identified MYC as a plausible driver gene and a central regulator of malignant transformation in initial stages of hepatocarcinogenesis (126). In primary and advanced HCC and in derived cell lines, MYC is commonly overexpressed because of genomic amplification. Using a conventional CGH analysis in HCC cell lines, we detected a DNA copy number increase throughout the 8q arm, and the minimal common region of amplification confined to band 8q24.1, which corresponds with the site of FRA8C and with location of MYC locus (12). In this study and in several others, the CGH analyses did not establish a correlation between genomic imbalances and the etiology of HCC (127). It is interesting that a CGH analysis and gene expression profiling established both the copy-number gains and MYC overexpression in viral and alcohol-related HCC, but not in cryptogenic, nonalcoholic, steatohepatitis-induced HCC (128).

In an earlier minireview article, describing chromosome-mediated alterations of MYC in various cancers, we discussed the impact of viral integration in hepatocarcinogenesis, and stressed the finding that the woodchuck hepatitis virus (WHV) is known to integrate near MYC locus and cause enhanced MYC expression (114). HBV has a genomic structure similar to that of WHV, and both viruses exhibit similarities to retroviruses in terms of integration and requirements of RNA intermediate and reverse transcriptase for replication. It is possible that HBV integrates at FRA8C and activates the MYC gene (129). However, due to HBV’s capacity to cause secondary genomic rearrangements and virus relocation, the localization of HBV integration sites in HCC may not represent the initial site of integration (114). Protooncogenes other than MYC often are amplified in HCC because of HBV integration (130). Mutagens-carcinogens also target FSs and induce genomic and gene alterations (131). It is possible that damage inflicted at FRA8C may explain the high frequency of MYC overexpression in viral and alcohol-related HCC (128), in addition to the ability of HBV’s X protein to produce such an effect (132).

The development of mouse models for HCC provided an ideal experimental approach for the detection of cellular and genetic alterations and the discovery of candidate cancer genes relevant to human hepatocarcinogenesis. Several new mouse models for HCC have been developed in our laboratory, among which MYC single-, and MYC-TGFα double-transgenic mice underscore the critical role of the MYC gene in hepatocarcinogenesis (133).

Such models offer the opportunity to detect lesions associated with the initial stages of neoplastic development, and identify alterations related to the acquisition of malignancy or to tumor progression. A conventional cytogenetic analysis revealed that at 10 weeks of age, the level of structural chromosomal aberrations was nearly tenfold higher in MYC-TGFα hepatocytes than in wild-type hepatocytes, and that, as a sign of genomic instability, early displastic lesions display breakage on several chromosomes, whose locations correspond with regions of tumor susceptibility (134). Also, it has been postulated that increased generation of reactive oxygen species might be responsible for the extensive chromosomal damage and acceleration of hepatocarcinogenesis characteristic of MYC-TGFα mice.

To determine whether vitamin E (VE), a potent anti-oxidant, is able to protect against the chromosomal damage triggered by oxidative stress caused by overexpression of c-myc and TGF-α transgenes, the incidence of chromosomal and chromatid aberrations was examined in primary hepatocyte cultures established from 10-week-old MYC-TGFα mice maintained on either a control diet, or a VE-supplemented diet. VE supplementation markedly decreased the frequency of chromosomal damage at the early stage of hepatocarcinogenesis before the development of morphologically defined preneoplastic and neoplastic lesions (135). The cytogenetic constitution of tumors developed in MYC-TGFα transgenic mice has been analyzed using conventional G-banding analysis and FISH with several chromosome painting and single-copy gene probes. Cells from all such cytogenetically characterized tumors maintained ability to form tumors after reinoculation in nude mice. The most frequently observed stable alterations involved chromosomes 1, 4, 6, 7, and 12. A correspondence existed between the location of breakpoints in stable alterations of these chromosomes, and the regions of fragility identified in the early stage of hepatocarcinogenesis, thus strongly suggesting the importance of these nonrandom changes in both initiation and progression of neoplasia (136). The location of MYC transgene was identified at chromosome 5G-ter; interestingly, in all tumors and derivative HCC cell lines, a balanced translocation t(5;6) was identified, with the breakpoint near the site of MYC transgene integration. This is the first balanced translocation found in either human or mouse liver tumors (137,138). Significantly, near the balanced translocation t(5;6), we characterized a deletion that inactivated a gene whose human homolog, GTF2IRD1, encodes a widely expressed, multifunctional helix loop helix transcription factor that binds to pRb and is one of the 16 genes present in approximately 1.6 Mb interval commonly deleted in Williams-Beuren syndrome (WBS) (139). Therefore, the c-myc transgenic mice carrying the t(5;6) translocation represent, also, the first ‘knockout’ of one of the genes present in the WBS critical region. The Gtf2ird1-null mice exhibited growth retardation and craniofacial and neurologic abnormalities similar to clinical symptoms in WBS patients (140).

Our combined SKY and aCGH analysis of several cell lines derived from HCC developed spontaneously in MYC transgenic mice, identified recurrent chromosome rearrangements and genomic imbalances. Among genomic imbalances, partial or complete gain of chromosomes 15 and 19 and loss of chromosomes 4, 9, and 14 were the most common alterations. These alterations are also recurrent in HCC developed in other transgenic mouse models, in mouse spontaneous HCC, in derivative cell lines, and in preneoplastic liver lesions induced by chemical carcinogens. Overall, these results demonstrate selective, nonrandom genomic changes associated with development of HCC (141).

Among those changes, gains of chromosomes 15 and 19 are particularly important. Mouse chromosome 15 bears large regions of homology with human chromosome 8q, and it is likely that increased copy number of chromosome 15 might enhance the effect of MYC in development of HCC, by providing more copies of the gene. DM and HSR have been found in a variety of solid tumors and hematologic malignancies but not in adult HCC or in HCC developed in animals (137,138). In a cell line derived from HCC developed in MYC transgenic mice, we detected a recurrent gain of chromosome 19 and DM derived from chromosome 19. At the site of DNA amplification are located the MYCS and MXI1 genes, both known to interfere with MYC gene activity, and the mouse RelA gene that promotes cell survival by inhibiting apoptosis and allowing MYC to drive proliferation of transformed cells (142).

Other related studies demonstrated that coexpression of MYC and TGF-α enhances the development of HCC through the disruption of the pRb/EF2 pathway and that TGF-α might function as a survival factor for neoplastic cells, thereby accelerating the neoplastic process (143). In a different mouse model, it has been demonstrated that MYC inactivation induced regression of invasive HCC resulting in differentiation into hepatocytes and biliary cells forming bile duct structures. This suppressive effect was accompanied by a loss of expression of the tumor markers and an increase in expression of liver cell and liver stem cell markers (144).

## 9. Modulation of DLC1 and MYC in hepatocellular carcinoma: prospects for combined pharmacologic interventions

There is no question that downregulation or silencing of DLC1 gene and/or amplification or upregulation of MYC gene are major contributing factors in the pathogenesis of human and murine HCC. A highly recurrent pattern of genomic imbalances in HCC predicts that both DLC1 and MYC would be deregulated in a large number of cases and that such synchronyzation may be consequential in hepatocarcinogenesis. This was demonstrated in an *in vitro*/*in vivo* reconstitution mouse model for HCC (53). In this model, genetically modified liver progenitor cells lacking p53, transduced with a retrovirus expressing MYC and with another expressing a DLC1 shRNA, were transplanted into the liver of syngeneic mice (53,145). DLC1 knockdown aided MYC in the induction of HCC in mice, and tumors thus developed are similar to aggressive human HCC. Ectopic overexpression of DLC1 in HCC cells expressing oncogenic Ras and containing increased GTP-bound RhoA, abolished the tumor formation. Conversely, suppression of RhoA inhibited growth of DLC1-negative HCC cells, showing that loss of DLC1 and RhoA activation has a similar effect in hepatocarcinogenesis. The results of this study provide conclusive evidence that loss of DLC1, combined with an oncogenic stimulus, promotes HCC *in vivo* and that this oncogenic process is associated with activation of RhoA, which is a key consequence of loss of DLC1 tumor suppressor activity (53,54).

Given the fact that many types of cancer accumulate multiple genetic alterations over time, the treatment of cancer by targeting a single damaged gene was originally considered unlikely to be effective. However, numerous experiments subsequently showed that silencing of an oncogene or activation of a TSG can be sufficient to abolish cell tumorigenicity (146). Despite the accumulation of a multitude of genetic alterations, tumor cells may depend only on a single oncogenic pathway to provide critical competitive advantage for their survival and unlimited multiplication. This phenomenon, known as ‘oncogene addiction,’ was described more than a decade ago (147). Only in the past few years, however, has this concept received renewed interest, and its potential to translate into effective targeted cancer therapy and the need for future investigations have been eloquently advocated (148). Suppression of such a critical oncogenic pathway may result in the death of tumor cells without any harmful consequences in normal cells. Likewise, reintroduction of the wild-type of TSGs, which are frequently inactivated in cancer cells, referred to as ‘tumor suppressor hypersensitivity,’ prevents tumor growth by inducing cell death or cell cycle arrest, and in many instances, it yields a complete response without toxicity to normal cells. These are fundamental requirements for an efficient cancer therapy.

It is well documented that DLC1 and MYC are deregulated in a large fraction of HCC. In a preclinical study designed to provide ground for potential tailored therapeutics, transplanting tumor tissue from HCC patients into nude mice has generated a number of explant models. The analyses of gene copy number, gene mutation, mRNA expression, and protein expression profiles demonstrated that transplanted tumors preserve the genotypic alterations of the original tumors. Consistent with the concept of oncogene addiction and tumor suppressor hypersensitivity, amplification of MYC and cyclin D1 oncogenes and deletion of DLC1 and FHIT TSGs were detected in different individual models, as well as in corresponding patients’ HCC tissues (149).

Novel therapeutic intervention options targeting the DLC1 pathway in HCC have been discussed and the clinical therapeutic efficacy of various agents was rigorously evaluated in several articles (53,54,85,150). Therapeutic interventions based on DLC1 activity require a full understanding of signaling pathways affected by loss of its function (54). The inhibition of RhoA pathway and Rho kinase (ROCK), a downstream effector of Rho, tops the options for therapeutic interventions (53,54,85,150). Currently, a major effort is underway to develop small molecule Rho kinase inhibitors to treat various disorders, including cancer. An effective therapeutic agent, Y-27632 displays distinct antimetastatic activity in HCC (151,152). Wf-536, a more potent derivative of Y-27632, also inhibited the metastasis of melanoma *in vivo* but has not yet been tested in HCC (153,154). Results with a new potent and selective ROCK inhibitor that is superior to Y compounds will be reported soon (Channing Der, personal communication).

A number of compounds that restore DLC1 expression, extend the half-life of its protein, or mimic its function, in different cancers, also might be effective in therapy for HCC. The ability of flavone to restore DLC1 expression and, consequently, to suppress metastatic breast cancer cell proliferation is not an isolated example (155). Other agents, such as all-trans retinoic acid and peroxisome proliferator-activated receptor γ (PPARγ), significantly elevated DLC1 expression in cancer cells (50). Morellofalvone, a biflavonoid, also inhibited tumor growth and angiogenesis of prostate cancer xenografts in mice by targeting RhoA and Rac1 GTPases (156). A newly developed synthetic flavone derivative, 6f, induces apoptosis mediated through death receptor and mitochondria-dependent pathway in human HCC (157). Thiazolidinedione, a synthetic activator of PPARγ, inhibits the growth of PPARg-expressing human colon cancer cells by inducing terminal differentiation and a marked increase in p21 abundance (158). Because epigenetic modifications are the main cause of DLC1 down-regulation and inactivation, therapy based on the ability of DNA methyl-transferase and histone deacetylase inhibitors (HDAC) to restore DLC1 expression in cancer cells has been highlighted as an attractive therapeutic approach (54). In HCC cells, a combined treatment involving suberoylanilide hydroxamic acid, a powerful HDAC inhibitor already used in clinical trials, and DLC1 transduction had a synergistic inhibitory effect on tumor cell proliferation and anchorage-independent growth (159). Other unrelated agents, such as ursodeoxycholic acid (UDCA), a hydrophilic bile acid, inhibited *in vitro* HCC cell proliferation by increasing the half-life of DLC1 protein and reducing RhoA activity (160). It remains to be seen whether UDCA has a similar antiproliferative effect in xenografted HCC in mice.

It is disappointing that after many years of intense effort and despite major advances in understanding the regulation and function of the MYC gene, the development of cancer therapeutics that would target MYC itself remains elusive (111). Future pharmacologic interventions targeting MYC should be guided by a new strategy that takes into account the differential MYC roles of either tumor promoting or tumor suppressing, depending on its level of expression, and also should consider modulation of the two opposite functions of MYC oncoprotein (161,162).

An informative review article outlined the modest progress in development of MYC targeted therapeutics in HCC. For example, small MYC inhibitors showed an antiproliferative effect on HCC cells *in vitro* and, furthermore, sensitized those cells to chemotherapeutic agents, but they were not equally effective *in vivo*(163). Quarfloxin CX-3453, which targets MYC expression through a four-stranded DNA structure, reached clinical trials for neuroendocrine carcinoma (164). Overall, there are few treatment options for advanced HCC. The development of Sorafenib, a multikinase inhibitor that marked a breakthrough in understanding of the disease, has shown remarkable survival benefits in patients with advanced HCC (165,166). Quarfloxin has the potential to be effective in therapy for HCC because, like Sorafenib, it inhibits vascular endothelial growth factor or may enhance the antitumor effect of Sorafenib (163).

Numerous studies suggest that statins, the cholesterol-lowering drugs, have an antiproliferative effect on cancer cells. Significantly, an interesting recent study demonstrated that inhibition of HMG-coenzyme A reductase by Atorvastatin blocks MYC phosphorylation and activation, leading to suppression of mouse and human HCC cell proliferation *in vivo* and *in vitro*. Suppression of MYC phosphorylation was, most likely, mediated through the inhibition of GTPases in the Rac pathway *in vitro*(167). In a clinical trial, treatment with Pravastatin improved survival in patients with HCC (168). Regarding the effects of statins, there is a significant link with DLC1 gene, which in its COOH-terminal regions harbors a START domain, thought to be important in cholesterol binding (36,169,170). As discussed earlier, Cav1, a cholesterol transporter, interacts with DLC1 START domain and contributes to the tumor suppressor activity of DLC1 (101). It is possible that statins may affect normal START function.

In summary, given the limitations of single-agent therapy, a combination of molecular therapies that target different pathways is expected to be more effective in treatment of HCC (166). Cancer therapeutics targeting gain and loss of function of oncogenes and TSGs, respectively, could lead to the identification of novel genotype-selective antitumor agents (171). In that respect, a combined therapeutic approach, targeting both MYC and DLC1 signaling networks, have realistic potential to improve the treatment of liver cancer.

## Figures and Tables

**Table I:** 

A, Candidate or bonafide tumor suppressor genes on chromosome 8p		

Name	Location	Type of cancer
MSRA	p23	Liver
**DLC1**	p22	Liver and other cancers
N33	p22	Prostate
PDGFRL	p22	Liver
MTUS1	p22	Liver and other cancers
LFIRE-1	p22	Liver
LZTS1	p22	Various cancers
TRAIL-R1	p21	Various cancers
TRAIL-R2	p21	Various cancers
DBC2	p21	Breast
RHOBTB2	p21	Breast
DOK	p21	Lung
SORBS3	p21	Liver
SHRBS3	p21	Liver
SCARA5	p12-11.1	Liver
NRG1	p11-12	Breast, pancreas
PROSC	p11	Liver
FGFR	p11	Myeloproliferative syndrome
TACC	p11	Breast
SFRP1	p11	Liver, breast
MOZ	p11	Acute myeloid leukemia

B, Protooncogenes on chromosome 8q		

Name	Location	Type of cancer
MOS	q11	Lung and other cancers
MDTH	q22.1	Breast
**MYC**	q24	Liver and other cancers
HSF1	q24.3	Liver
LYN	q13-qter	Ewing’s sarcoma

## References

[1] Hanahan D, Weinberg RA (2011). Hallmarks of cancer: the next generation. Cell.

[2] Bishop JM (1987). The molecular genetics of cancer. Science.

[3] Rowley JD (1980). Ph1-positive leukaemia, including chronic myelogenous leukaemia. Clin Haematol.

[4] Kraus MH, Popescu NC, Amsbaugh SC, King CR (1987). Overexpression of the EGF receptor-related protooncogene erbB-2 in human mammary tumor cell lines by different molecular mechanisms. EMBO J.

[5] Jones PA, Baylin SB (2007). The epigenomics of cancer. Cell.

[6] Gore SD, Baylin S, Sugar E, Carraway H, Miller CB, Carducci M, Grever M, Galm O, Dauses T, Karp JE, Rudek MA, Zhao M, Smith BD, Manning J, Jiemjit A, Dover G, Mays A, Zwiebel J, Murgo A, Weng LJ, Herman JG (2006). Combined DNA methyltransferase and histone deacetylase inhibition in the treatment of myeloid neoplasms. Cancer Res.

[7] Richon VM, Garcia-Vargas J, Hardwick JS (2009). Development of vorinostat: current applications and future perspectives for cancer therapy. Cancer Lett.

[8] Thorgeirsson SS, Grisham JW (2002). Molecular pathogenesis of human hepatocellullar carcinoma. Nat Genet.

[9] Altekruse SF, McGlynn KA, Reichman ME (2009). Hepatocellular carcinima incidence, mortality and survival trends in United States from 1975 to 2005. J Clin Oncol.

[10] Jones RG, Thompson CB (2009). Tumor suppressor and cell metabolism: a recipe for cancer growth. Genes Dev.

[11] Keck CL, Zimonjic DB, Yuan BZ, Thorgeirsson SS, Popescu NC (1999). Nonrandom breakpoints of unbalanced chromosome translocations in human hepatocellular carcinoma cell lines. Cancer Genet Cytogenet.

[12] Zimonjic DB, Keck CL, Thorgeirsson SS, Popescu NC (1999). Novel recurrent genetic imbalances in human hepatocellular carcinoma cell lines identified by comparative genomic hybridization. Hepatology.

[13] Popescu NC (2003). Genetic alterations in cancer as a result of breakage at fragile sites. Cancer Lett.

[14] Garzon R, Calin GA, Croce CM (2009). MicroRNAs in cancer. Annu Rev Med.

[15] Simon D, Knowles B, Weith A (1991). Abnormalities of chromosome 1 and loss of heterozygosity on 1p in primary hepatomas. Oncogene.

[16] Yeh SH, Chen PJ, Chen HL, Lai MY, Wang CC, Chen DS (1994). Frequent genetic alterations at the distal region of chromosome 1p in human hepatocellular carcinomas. Cancer Res.

[17] Woo HG, Park ES, Lee JS, Lee YH, Ishikawa T, Kim YJ, Thorgeirsson SS (2009). Identification of potential driver genes in human liver carcinoma by genomewide screening. Cancer Res.

[18] Yuan BZ, Keck-Waggoner C, Zimonjic DB, Thorgeirsson SS, Popescu NC (2000). Alterations of FHIT gene in human hepatocellular carcinoma. Cancer Res.

[19] Imreh S, Klein G, Zabarovsky ER (2003). Search for unknown tumor-antagonizing genes. Genes Chromosomes Cancer.

[20] Zhou X, Popescu NC, Klein G, Imreh S (2007). The interferon-alpha responsive gene TMEM7 suppresses cell proliferation and is downregulated in human hepatocellular carcinoma. Cancer Genet Cytogenet.

[21] Zimonjic DB, Zhou X, Lee JS, Ullmannova-Benson V, Tripathi V, Thorgeirsson SS, Popescu NC (2009). Acquired genetic and functional alterations associated with transforming growthfactor beta type I resistance in Hep3B human hepatocellular carcinoma cell line. J Cell Mol Med.

[22] Ludes-Meyers JH, Bednarek AK, Popescu NC, Bedford M, Aldaz CM (2003). WWOX, the common chromosomal fragile site, FRA16D, cancer gene. Cytogenet Genome Res.

[23] Park SW, Ludes-Meyers J, Zimonjic DB, Durkin ME, Popescu NC, Aldaz CM (2004). Frequent downregulation and loss of WWOX gene expression in human hepatocellular carcinoma. Br J Cancer.

[24] Yuan BZ, Zhou X, Zimonjic DB, Durkin ME, Popescu NC (2003). Amplification and overexpression of the EMS 1 oncogene, a possible prognostic marker, in human hepatocellular carcinoma. J Mol Diagn.

[25] Zimonjic DB, Durkin ME, Keck-Waggoner CL, Park SW, Thorgeirsson SS, Popescu NC (2003). SMAD5 gene expression, re arrangements, copy number, and amplification at fragile site FRA5C in human hepatocellular carcinoma. Neoplasia.

[26] Emi M, Fujiwara Y, Ohata H, Tsuda H, Hirohashi S, Koike M, Miyaki M, Monden M, Nakamura Y (1993). Allelic loss at chromosome band 8p21.3-p22 is associated with progression of hepatocellular carcinoma. Genes Chromosomes Cancer.

[27] Pineau P, Nagai H, Prigent S, Wei Y, Gyapay G, Weissenbach J, Tiollais P, Buendia MA, Dejean A (1999). Identification of three distinct regions of allelic deletions on the short arm of chromosome 8 in hepatocellular carcinoma. Oncogene.

[28] Qin LX, Tang ZY, Sham JS, Ma ZC, Ye SL, Zhou XD, Wu ZQ, Trent JM, Guan XY (1999). The association of chromosome 8p deletion and tumor metastasis in human hepatocellular carcinoma. Cancer Res.

[29] Chan KL, Lee JM, Guan XY, Fan ST, Ng IO (2002). High-density allelotyping of chromosome 8p in hepatocellular carcinoma and clinicopathologic correlation. Cancer.

[30] Kahng YS, Lee YS, Kim BK, Park WS, Lee JY, Kang CS (2003). Loss of heterozygosity of chromosome 8p and 11p in the dysplastic nodule and hepatocellular carcinoma. J Gastroenterol Hepatol.

[31] Pang JZ, Qin LX, Ren N, Hei ZY, Ye QH, Jia WD, Sun BS, Lin GL, Liu DY, Liu YK, Tang ZY (2007). Loss of heterozygosity at D8S298 is a predictor for long-term survival of patients with tumor-node-metastasis stage I of hepatocellular carcinoma. Clin Cancer Res.

[32] Yam JW, Wong CM, Ng IO (2010). Molecular and functional genetics in hepatocellular carcinoma. Front Biosci (Schol Ed).

[33] Birnbaum D, Adélaïde J, Popovici C, Charafe-Jauffret E, Mozziconacci MJ, Chaffanet M (2003). Chromosome arm 8p and cancer: a fragile hypothesis. Lancet Oncol.

[34] Popescu NC (2004). Fragile sites and cancer genes on the short arm of chromosome 8. Lancet Oncol.

[35] Fujiwara Y, Ohata H, Kuroki T, Koyama K, Tsuchiya E, Monden M, Nakamura Y (1995). Isolation of a candidate tumor suppressor gene on chromosome 8p21.3-p22 that is homologous to an extracellular domain of the PDGF receptor beta gene. Oncogene.

[36] Yuan BZ, Miller MJ, Keck CL, Zimonjic DB, Thorgeirsson SS, Popescu NC (1998). Cloning, characterization, and chromosomal localization of a gene frequently deleted in human liver cancer (DLC-1) homologous to rat RhoGAP. Cancer Res.

[37] Yan J, Yu Y, Wang N, Chang Y, Ying H, Liu W, He J, Li S, Jiang W, Li Y, Liu H, Wang H, Xu Y (2004). LFIRE-1/HFREP-1, a liver-specific gene, is frequently downregulated and has growth suppressor activity in hepatocellular carcinoma. Oncogene.

[38] Shih YL, Shyu RY, Hsieh CB, Lai HC, Liu KY, Chu TY, Lin YW (2006). Promoter methylation of the secreted frizzled-related protein 1 gene SFRP1 is frequent in hepatocellular carcinoma. Cancer.

[39] Huang J, Zhang YL, Teng XM, Lin Y, Zheng DL, Yang PY, Han ZG (2007). Down-regulation of SFRP1 as a putative tumor suppressor gene can contribute to human hepatocellular carcinoma. BMC Cancer.

[40] Lei KF, Wang YF, Zhu XQ, Lu PC, Sun BS, Jia HL, Ren N, Ye QH, Sun HC, Wang L, Tang ZY, Qin LX (2007). Identification of MSRA gene on chromosome 8p as a candidate metastasis suppressor for human hepatitis B virus-positive hepatocellular carcinoma. BMC Cancer.

[41] Huang J, Zheng DL, Qin FS, Cheng N, Chen H, Wan BB, Wang YP, Xiao HS, Han ZG (2010). Genetic and epigenetic silencing of SCARA5 may contribute to human hepatocellular carcinoma by activating FAK signaling. J Clin Invest.

[42] Finch PW, He X, Kelley MJ, Uren A, Schaudies P, Popescu NC, Rudicoff S, Aaronson SA, Varmus HE, Rubin JS (1997). Purification and molecular cloning of a secreted, Frizzled-related antagonist of Wnt action. Proc Natl Acad Sci USA.

[43] Rubin JS, Barshishat-Kupper M, Feroze-Merzoug F, Xi ZF (2006). Secreted WNT antagonists as tumor suppressors: pro and con. Front Biosci.

[44] Saini S, Liu J, Yamamura S, Majid S, Kawakami K, Hirata H, Dahiya R (2009). Functional significance of secreted Frizzled-related protein 1 in metastatic renal cell carcinomas. Cancer Res.

[45] Kawamoto K, Hirata H, Kikuno N, Tanaka Y, Nakagawa M, Dahiya R (2008). DNA methylation and histone modifications cause silencing of Wnt antagonist gene in human renal cell carcinoma cell lines. Int J Cancer.

[46] Thompson MD, Monga SP (2007). WNT/beta-catenin signaling in liver health and disease. Hepatology.

[47] Takigawa Y, Brown AM (2008). Wnt signaling in liver cancer. Curr Drug Targets.

[48] Ching YP, Wong CM, Chan SF, Leung TH, Ng DC, Jin DY, Ng IO (2003). Deleted in liver cancer (DLC) 2 encodes a RhoGAP protein with growth suppressor function and is underexpressed in hepatocellular carcinoma. J Biol Chem.

[49] Durkin EM, Ullmannova V, Guan M, Popescu NC (2007). Deleted in liver cancer 3(DLC-3), a novel RhoGTPase-activating protein, is downregulated in cancer and inhibits tumor cell growth. Oncogene.

[50] Durkin ME, Yuan BZ, Zhou X, Zimonjic DB, Lowy DR, Thorgeirsson SS, Popescu NC (2007). DLC-1: a Rho GTPase-activating protein and tumor suppressor. J Cell Mol Med.

[51] Low JS, Tao Q, Ng KM, Goh HK, Shu XS, Woo WL, Ambinder RF, Srivastava G, Shamay M, Chan AT, Popescu NC, Hsieh WS (2011). A novel isoform of the 8p22 tumor suppressor gene DLC1 suppresses tumor growth and is frequently silenced in multiple common tumors. Oncogene.

[52] Liao YC, Lo SH (2008). Deleted in liver cancer-1 (DLC-1): a tumor suppressor not just for liver. Int J Biochem Cell Biol.

[53] Xue W, Krasnitz A, Lucito R, Sordella R, Vanaelst L, Cordon-Cardo C, Singer S, Kuehnel F, Wigler M, Powers S, Zender L, Lowe SW (2008). DLC1 is a chromosome 8p tumor suppressor whose loss promotes hepatocellular carcinoma. Genes Dev.

[54] Lahoz A, Hall A (2008). DLC1: a significant GAP in the cancer genome. Genes Dev.

[55] Vigil D, Cherfils J, Rossman KL, Der CJ (2010). Ras superfamily GEFs and GAPs: validated and tractable targets for cancer therapy?. Nat Rev Cancer.

[56] Durkin ME, Avner MR, Huh CG, Yuan BZ, Thorgeirsson SS, Popescu NC (2005). DLC-1, a Rho GTPase-activating protein with tumor suppressor function, is essential for embryonic development. FEBS Lett.

[57] Hers I, Wherlock M, Homma Y, Yagisawa H, Tavaré JM (2006). Identification of p122RhoGAP (deleted in liver cancer-1) Serine 322 as a substrate for protein kinase B and ribosomal S6 kinase in insulin-stimulated cells. J Biol Chem.

[58] Murakami R, Osanai T, Tomita H, Sasaki S, Maruyama A, Itoh K, Homma Y, Okumura K (2010). p122 protein enhances intra-cellular calcium increase to acetylcholine: its possible role in the pathogenesis of coronary spastic angina. Arterioscler Thromb Vasc Biol.

[59] Wu J, Li Y, Fan X, Zhang C, Wang Y, Zhao Z (2011). Analysis of gene expression profile of periodontal ligament cells subjected to cyclic compressive force. DNA Cell Biol.

[60] Ng IO, Liang ZD, Cao L, Lee TK (2000). DLC1 is deleted in primary hepatocellular carcinoma and exerts inhibitory effects on the proliferation of hepatoma cell lines with deleted DLC1. Cancer Res.

[61] Park SW, Durkin ME, Thorgeirsson SS, Popescu NC (2003). DNA variants of DLC-1, a candidate tumor suppressor gene in human hepatocellular carcinoma. Int J Oncol.

[62] Liao YC, Shih YP, Lo SH (2008). Mutations in the focal adhesion targeting region of deleted in liver cancer-1 attenuate their expression and function. Cancer Res.

[63] Jones S, Zhang X, Parsons DW, Lin JC, Leary RJ, Angenendt P, Mankoo P, Carter H, Kamiyama H, Jimeno A, Hong SM, Fu B, Lin MT, Calhoun ES, Kamiyama M, Walter K, Nikolskaya T, Nikolsky Y, Hartigan J, Smith DR, Hidalgo M, Leach SD, Klein AP, Jaffee EM, Goggins M, Maitra A, Iacobuzio-Donahue C, Eshleman JR, Kern SE, Hruban RH, Karchin R, Papadopoulos N, Parmigiani G, Vogelstein B, Velculescu VE, Kinzler KW (2008). Core signaling pathways in human pancreatic cancers revealed by global genomic analyses. Science.

[64] Yachida S, Jones S, Bozic I, Antal T, Leary R, Fu B, Kamiyama M, Hruban RH, Eshleman JR, Nowak MA, Velculescu VE, Kinzler KW, Vogelstein B, Iacobuzio- Donahue CA (2010). Distant metastasis occurs late during the genetic evolution of pancreatic cancer. Nature.

[65] Dong X, Zhou G, Zhai Y, Zhang H, Yang H, Zhi L, Zhang X, Chu J, He F (2009). Association of DLC1 gene polymorphism with susceptibility to hepatocellular carcinoma in Chinese hepatitis B virus carriers. Cancer Epidemiol.

[66] Teodoridis JM, Hardie C, Brown R (2008). CpG island methylator phenotype (CIMP) in cancer: causes and implications. Cancer Lett.

[67] Yuan BZ, Durkin ME, Popescu NC (2003). Promoter hypermethylation of DLC-1, a candidate tumor suppressor gene, in several common human cancers. Cancer Genet Cytogenet.

[68] Wong CM, Lee JM, Ching YP, Jin DY, Ng IO (2003). Genetic and epigenetic alterations of DLC-1 gene in hepatocellular carcinoma. Cancer Res.

[69] Ko FC, Yeung YS, Wong CM, Chan LK, Poon RT, Ng IO, Yam JW (2010). Deleted in liver cancer 1 isoforms are distinctly expressed in human tissues, functionally different and under differential transcriptional regulation in hepatocellular carcinoma. Liver Int.

[70] Croce MC (2009). Causes and consequences of microRNA dysregulation in cancer. Nature Rev Genet.

[71] Banaudha K, Kaliszewski M, Korolnek T, Florea L, Yeung ML, Kuan KT, Kumar A (2011). MicroRNA silencing of tumor suppressor DLC-1 promotes efficient hepatitis C virus replication in primary human hepatocytes. Hepatology.

[72] Wong CM, Yam JW, Ching YP, Yau TO, Leung TH, Jin DY, Ng IO (2005). Rho GTPase-activating protein deleted in liver cancer suppresses cell proliferation and invasion in hepatocellular carcinoma. Cancer Res.

[73] Healy KD, Hodgson L, Kim TY, Shutes AT, Maddileti S, Juliano RL, Hahn KM, Harden TK, Bang YJ, Der CJ (2008). DLC1 suppresses non-small lung cancer growth and invasion by RhoGAP-dependent and independent mechanisms. Mol Carcinog.

[74] Kim TY, Lee JW, Kim HP, Jong HS, Kim TY, Jung M, Bang YJ (2007). DLC-1, a GTPase-activating protein for Rho, is associated with cell proliferation, morphology and migration in human hepatocellular carcinoma. Biochem Biophys Res Commun.

[75] Qian X, Li G, Asmussen HK, Asnaghi L, Vass WC, Braverman R, Yamada KM, Popescu NC, Papageorge AG, Lowy DR (2007). Oncogenic inhibition by a deleted in liver cancer gene requires cooperation between tensin binding and Rho-specific GTPase-activating protein activities. Proc Natl Acad Sci USA.

[76] Guan M, Tripathi V, Zhou X, Popescu NC (2008). Adenovirus-mediated restoration of the expression of the tumor suppressor gene DLC1 inhibits the proliferation and tumorigenicity of aggressive, androgen-independent human prostate cancer cell lines: Prospects for gene therapy. Cancer Gene Ther.

[77] Zhou X, Zimonjic DB, Park SW, Yang XY, Durkin ME, Popescu NC (2008). DLC1 suppresses distant dissemination of human hepatocellular carcinoma cells in nude mice through reduction of RhoA GTPase activity, actin cytoskeletal disruption and down-regulation of genes involved in metastasis. Int J Oncol.

[78] Holeiter G, Heering J, Erlmann P, Schmid S, Jähne R, Olayioye MA (2008). Deleted in liver cancer 1 controls migration through a Dia1-dependent signaling pathway. Cancer Res.

[79] Erlmann P, Schmid S, Horenkamp FA, Geyer M, Pomorski TG, Olayioye M (2009). DLC1 activation requires lipid interation through a polybasic region preceding the RhoGap domain. Mol Biol Cell.

[80] Zhong D, Zhang J, Yang S, Soh UJ, Buschdorf JP, Zhou YT, Yang D, Low BC (2009). The SAM domain of the RhoGAP DLC1 binds EF1A1 to regulate cell migration. J Cell Sci.

[81] Sahai E, Marshall CJ (2002). RHO-GTPases and cancer. Nat Rev Cancer.

[82] Gómez del Pulgar T, Benitah SA, Valerón PF, Espina C, Lacal JC (2005). Rho GTPase expression in tumourigenesis: evidence for a significant link. Bioessays.

[83] Jaffe AB, Hall A (2002). Rho GTPases in transformation and metastasis. Adv Cancer Res.

[84] Ridley AJ (2004). Rho proteins and cancer. Breast Cancer Res Treat.

[85] Grise F, Bidaud A, Moreau V (2009). Rho GTPases in hepatocellular carcinoma. Biochim Biophys Acta.

[86] Roessler S, Long EL, Budhu A, Chen Y, Zhao X, Ji J, Walker R, Jia HL, Ye QH, Qin LX, Tang ZY, He P, Hunter KW, Thorgeirsson SS, Meltzer PS, Wang XW (2011). Integrative genomic identification of genes on 8p associated with hepatocellular carcinoma progression and patient survival. Gastroenterology.

[87] Pihur V, Datta S, Datta S (2008). Finding common genes in multiple cancer types through meta-analysis of microarray experiments: a rank aggregation approach. Genomics.

[88] Zhou X, Thorgeirsson SS, Popescu NC (2004). Restoration of DLC-1 gene expression induces apoptosis and inhibits both cell growth and tumorigenicity in human hepatocellular carcinoma cells. Oncogene.

[89] Kawai K, Yamaga M, Iwamae Y, Kiyota M, Kamata H, Hirata H, Homma Y, Yagisawa H (2004). A PLCdelta1-binding protein, p122RhoGAP, is localized in focal adhesions. Biochem Soc Trans.

[90] Kim TY, Vigil D, Der CJ, Juliano RL (2009). Role of DLC-1, a tumor suppressor protein with RhoGAP activity, in regulation of the cytoskeleton and cell motility. Cancer Metastasis Rev.

[91] Yuan BZ, Jefferson AM, Millecchia L, Popescu NC, Reynolds SH (2007). Morphological changes and nuclear translocation of DLC1 tumor suppressor protein precede apoptosis in human non-small cell lung carcinoma cells. Exp Cell Res.

[92] Goodison S, Yuan J, Sloan D, Kim R, Li C, Popescu NC, Urquidi V (2005). The RhoGAP protein DLC-1 functions as a metastasis suppressor in breast cancer cells. Cancer Res.

[93] Ko FC, Chan LK, Tung EK, Lowe SW, Ng IO, Yam JW (2010). Akt phosphorylation of deleted in liver cancer 1 abrogates its suppression of liver cancer tumorigenesis and metastasis. Gastroenterology.

[94] Yam JW, Ko FC, Chan CY, Jin DY, Ng IO (2006). Interaction of deleted in liver cancer 1 with tensin2 in caveolae and implications in tumor suppression. Cancer Res.

[95] Liao YC, Si L, deVere White RW, Lo SH (2007). The phosphotyrosine-independent interaction of DLC-1 and the SH2 domain of cten regulates focal adhesion localization and growth suppression activity of DLC-1. J Cell Biol.

[96] Hall EH, Daugherty AE, Choi CK, Horwitz AF, Brautigan DL (2009). Tensin1 requires protein phosphatase-1alpha in addition to RhoGAP DLC-1 to control cell polarization, migration, and invasion. J Biol Chem.

[97] Chan LK, Ko FC, Ng IO, Yam JW (2009). Deleted in liver cancer 1 (DLC1) utilizes a novel binding site for Tensin2 PTB domain interaction and is required for tumor-suppressive function. PLoS One.

[98] Hafizi S, Sernstad E, Swinny JD, Gomez MF, Dahlbäck B (2010). Individual domains of Tensin2 exhibit distinct subcellular localisations and migratory effects. Int J Biochem Cell Biol.

[99] Clark K, Howe JD, Pullar CE, Green JA, Artym VV, Yamada KM, Critchley DR (2010). Tensin 2 modulates cell contractility in 3D collagen gels through the RhoGAP DLC1. J Cell Biochem.

[100] Kawai K, Kitamura SY, Maehira K, Seike J, Yagisawa H (2010). START-GAP1/DLC1 is localized in focal adhesions through interaction with the PTB domain of tensin2. Adv Enzyme Regul.

[101] Du X, Qian X, Papageorge A, Vass WC, Braverman R, Lowy DR (2011). Complex formation between DLC START domain and Cav1 contributes to the tumor suppressor function of DLC1. Proc Am Assoc Cancer Res.

[102] Yang XY, Guan M, Vigil D, Der CJ, Lowy DR, Popescu NC (2009). p120Ras-GAP binds the DLC1 Rho-GAP tumor suppressor protein and inhibits its RhoA GTPase and growth-suppressing activities. Oncogene.

[103] Tripathi V, Zimonjic DB, Popescu NC (2011). DLC1 and α-catenin protein interaction enhances DLC1 antioncogenic activity by stabilizing adherens junctions and suppressing NFκB signaling. Proc Am Assoc Cancer Res.

[104] Yang X, Popescu NC, Zimonjic DB (2011). DLC1 interaction with S100A10 mediates inhibtion of *in vitro* cell invasion and tumorigenicity of lung cancer cells through a RhoGAP-indpendent mechanism. Cancer Res.

[105] Scholz RP, Gustafsson JO, Hoffmann P, Jaiswal M, Ahmadian MR, Eisler S, Erlmann P, Schmid S, Hausser A, Olayioye MA (2011). The tumor suppressor protein DLC1 is regulated by PKD-mediated GAP domain phosphorylation. Exp Cell Res.

[106] Tompa P (2002). Intrinsically unstructured proteins. Trends Biochem Sci.

[107] Hermeking H (2003). The 14-3-3 cancer connection. Nat Rev Cancer.

[108] Scholz RP, Regner J, Theil A, Erlmann P, Holeiter G, Jähne R, Schmid S, Hausser A, Olayioye MA (2009). DLC1 interacts with 14-3-3 proteins to inhibit RhoGAP activity and block nucleocytoplasmic shuttling. J Cell Sci.

[109] Wuestefeld T, Zender L (2010). DLC1 and liver cancer: the Akt connection. Gastroenterology.

[110] Oliveira AM, Ross JS, Fletcher JA (2005). Tumor suppressor genes in breast cancer: the gatekeepers and the caretakers. Am J Clin Pathol.

[111] Meyer N, Penn LZ (2008). Reflecting on 25 years with MYC. Nat Rev Cancer.

[112] Varmus H (1988). Retroviruses. Science.

[113] Peters G (1990). Oncogenes at viral integration sites. Cell Growth Differ.

[114] Popescu NC, Zimonjic DB (2002). Chromosome-mediated alterations of the MYC gene in human cancer. J Cell Mol Med.

[115] Payne GS, Bishop JM, Varmus HE (1982). Multiple arrangements of viral DNA and an activated host oncogene in bursal lymphomas. Nature.

[116] Popescu NC, Zimonjic DB, DiPaolo JA (1990). Viral integration, fragile sites and proto-oncogenes in human neoplasia. Hum Genet.

[117] Liu J, Kaur G, Zhawar VK, Zimonjic DB, Popescu NC, Kandpal R, Athwal RS (2009). Role of SV40 integration site at chromosomal interval 1q21.1 in immortalized CRL2504 cells. Cancer Res.

[118] Weinberg RA (1980). Integrated genomes of animal viruses. Annu Rev Biochem.

[119] Bester AC, Schwartz M, Schmidt M, Garrigue A, Hacein-Bey-Abina S, Cavazzana-Calvo M, Ben-Porat N, Von Kalle C, Fischer A, Kerem B (2006). Fragile sites are preferential targets for integrations of MLV vectors in gene therapy. Gene Ther.

[120] Croce CM, Nowell PC (1985). Molecular basis of human B cell neoplasia. Blood.

[121] Zimonjic DB, Keck-Waggoner C, Popescu NC (2001). Novel genomic imbalances and chromosome translocations involving c-myc gene in Burkitt’s lymphoma. Leukemia.

[122] Alitalo KM, Schwab M (1986). Oncogene amplification in tumor cells. Adv Cancer Res.

[123] Alitalo K, Schwab M, Lin CC, Varmus HE, Bishop JM (1983). Homogeneously staining chromosomal regions contain amplified copies of an abundantly expressed cellular oncogene (c-myc) in malignant neuroendocrine cells from a human colon carcinoma. Proc Natl Acad Sci USA.

[124] Zimonjic DB, Keck-Waggoner CL, Yuan BZ, Kraus MH, Popescu NC (2000). Profile of genetic alterations and tumorigenicity of human breast cancer cells. Int J Oncol.

[125] Elenbaas B, Spirio L, Koerner F, Fleming MD, Zimonjic DB, Donaher JL, Popescu NC, Hahn WC, Weinberg RA (2001). Human breast cancer cells generated by oncogenic transformation of primary mammary epithelial cells. Genes Dev.

[126] Kaposi-Novak P, Libbrecht L, Woo HG, Lee YH, Sears NC, Coulouarn C, Conner EA, Factor VM, Roskams T, Thorgeirsson SS (2009). Central role of c-Myc during malignant conversion in human hepatocarcinogenesis. Cancer Res.

[127] Farazi PA, DePinho RA (2006). Hepatocellular carcinoma pathogenesis: from genes to environment. Nat Rev Cancer.

[128] Schlaeger C, Longerich T, Schiller C, Bewerunge P, Mehrabi A, Toedt G, Kleeff J, Ehemann V, Eils R, Lichter P, Schirmacher P, Radlwimmer B (2008). Etiology-dependent molecular mechanisms in human hepatocarcinogenesis. Hepatology.

[129] Wei Y, Ponzetto A, Tiollais P, Buendia MA (1992). Multiple rearrangements and activated expression of c-myc induced by woodchuck hepatitis virus integration in a primary liver tumour. Res Virol.

[130] Tokino T, Matsubara K (1991). Chromosomal sites for hepatitis B virus integration in human hepatocellu lar carcinoma. J Virol.

[131] Yunis JJ, Soreng AL, Bowe AE (1987). Fragile sites are targets of diverse mutagens and carcinogens. Oncogene.

[132] Yang L, He J, Chen L, Wang G (2009). Hepatitis B virus X protein upregulates expression of SMYD3 and C-MYC in HepG2 cells. Med Oncol.

[133] Santoni-Rugiu E, Nagy P, Jensen MR, Factor VM, Thorgeirsson SS (1996). Evolution of neoplastic development in the liver of transgenic mice co-expressing c-myc and transforming growth factor. Am J Pathol.

[134] Sargent LM, Sanderson ND, Thorgeirsson SS (1996). Ploidy and karyotypic alterations associated with early events in the development of hepatocarcinogenesis in transgenic mice harboring c-myc and transforming growth factor alpha transgenes. Cancer Res.

[135] Factor VM, Laskowska D, Jensen MR, Woitach JT, Popescu NC, Thorgeirsson SS (2000). Vitamin E reduces chromosomal damage and inhibits hepatic tumor formation in a transgenic mouse model. Proc Natl Acad Sci USA.

[136] Sargent LM, Zhou X, Keck CL, Sanderson ND, Zimonjic DB, Popescu NC, Thorgeirsson SS (1999). Nonrandom cytogenetic alterations in hepatocellular carcinoma from transgenic mice overexpressing c-Myc and transforming growth factor-alpha in the liver. Am J Path.

[137] Grisham JW (1997). Interspecies comparison of liver carcinogenesis: implications for cancer risk assessment. Carcinogenesis.

[138] Grisham JW, Coleman WB, Tsongalis GT (2001). Molecular genetic alterations in primary hepatocellular meoplasm: hepatocellular adenoma, hepatocellular carcinoma, and hepatoblastoma. The Molecular Basis of Human Cancer.

[139] Durkin ME, Keck-Waggoner CL, Popescu NC, Thorgeirsson SS (2001). Integration of a c-myc transgene results in disruption of the mouse Gtf2ird1 gene, the homologue of the human GTF2IRD1 gene hemizygously deleted in Williams-Beuren syndrome. Genomics.

[140] Tassabehji M, Hammond P, Karmiloff-Smith A, Thompson P, Thorgeirsson SS, Durkin ME, Popescu NC, Hutton T, Metcalfe K, Rucka A, Stewart H, Read AP, Maconochie M, Donnai D (2005). GTF2IRD1 in craniofacial development of humans and mice. Science.

[141] Zimonjic DB, Ullmannova-Benson V, Factor VM, Thorgeirsson SS, Popescu NC (2009). Recurrent and nonrandom DNA copy number and chromosome alterations in Myc transgenic mouse model for hepatocellular carcinogenesis: implications for human disease. Cancer Genet Cytogenet.

[142] Zimonjic DB, Zhang H, Shan Z, Factor VM, Trent J, Thorgeirsson SS, Popescu NC (2001). DNA amplification associated with double minutes originating from chromosome 19 in mouse hepatocellular carcinoma. Cytogenet Cell Genet.

[143] Murakami H, Sanderson ND, Nagy P, Marino PA, Merlino G, Thorgeirsson SS (1993). Transgenic mouse model for synergistic effects of nuclear oncogenes and growth factors in tumorigenesis: interaction of c-myc and transforming growth factor alpha in hepatic oncogenesis. Cancer Res.

[144] Shachaf CM, Kopelman AM, Arvanitis C, Karlsson A, Beer S, Mandl S, Bachmann MH, Borowsky AD, Ruebner B, Cardiff RD, Yang Q, Bishop JM, Contag CH, Felsher DW (2004). MYC inactivation uncovers pluripotent differentiation and tumour dormancy in hepatocellular cancer. Nature.

[145] Zender L, Spector MS, Xue W, Flemming P, Cordon-Cardo C, Silke J, Fan ST, Luk JM, Wigler M, Hannon GJ, Mu D, Lucito R, Powers S, Lowe SW (2006). Identification and validation of oncogenes in liver cancer using an integrative oncogenomic approach. Cell.

[146] Roth JA, Grammer SF, El-Deiry WS (2003). Tumor suppressor gene therapy. Tumor Supressor Genes: Regulation, Function and Medicinal Applications.

[147] Weinstein IB (2002). Cancer. Addiction to oncogenes - the Achilles heel of cancer. Science.

[148] Sharma SV, Settleman J (2007). Oncogene addiction: setting the stage for molecularly targeted cancer therapy. Genes Dev.

[149] Zhang L, Zhang J, Xie L, Xie X, Guo Q, Lv J, Gao Z, Qian Z, Yin X, Zheng L, Zhu G, Ji Q, Ren Z (2011). Molecular characterization of hepatocellular carcinoma (HCC) patient derived explant models. Proc Am Assoc Cancer Res.

[150] Wong CC, Wong CM, Au SL, Ng IO (2010). RhoGTPases and Rho-effectors in hepatocellular carcinoma metastasis: ROCK N’ Rho move it. Liver Int.

[151] Takamura M, Sakamoto M, Genda T, Ichida T, Asakura H, Hirohashi S (2001). Inhibition of intrahepatic metastasis of human hepatocellular carcinoma by Rho-associated protein kinase inhibitor Y-27632. Hepatology.

[152] Ogawa T, Tashiro H, Miyata Y, Ushitora Y, Fudaba Y, Kobayashi T, Arihiro K, Okajima M, Asahara T (2007). Rho-associated kinase inhibitor reduces tumor recurrence after liver transplantation in a rat hepatoma model. Am J Transplant.

[153] Nakajima M, Hayashi K, Egi Y, Katayama K, Amano Y, Uehata M, Ohtsuki M, Fujii A, Oshita K, Kataoka H, Chiba K, Goto N, Kondo T (2003). Effect of Wf-536, a novel ROCK inhibitor, against metastasis of B16 melanoma. Cancer Chemother Pharmacol.

[154] McHenry PR, Vargo-Gogola T (2010). Pleiotropic functions of Rho GTPase signaling: a Trojan horse or Achilles’ heel for breast cancer treatment?. Curr Drug Targets.

[155] Ullmannova V, Popescu NC (2007). Inhibition of cell proliferation, induction of apoptosis, reactivation of DLC1, and modulation of other gene expression by dietary flavone in breast cancer cell lines. Cancer Detect Prev.

[156] Pang X, Yi T, Yi Z, Cho S G, Qu W, Pinkaew D, Fujise K, Liu M (2009). Morelloflavone, a biflavonoid, inhibits tumor angiogenesis by targeting rho GTPases and extracellular signal-regulated kinase signaling pathways. Cancer Res.

[157] Liu H, Dong A, Gao C, Tan C, Xie Z, Zu X, Qu L, Jiang Y (2010). New synthetic flavone derivatives induce apoptosis of hepatocarcinoma cells. Bioorg Med Chem.

[158] Yoshizumi T, Ohta T, Ninomiya I, Terada I, Fushida S, Fujimura T, Nishimura G, Shimizu K, Yi S, Miwa K (2004). Thiazolidinedione, a peroxisome proliferator-activated receptor-gamma ligand, inhibits growth and metastasis of HT-29 human colon cancer cells through differentiation-promoting effects. Int J Oncol.

[159] Zhou X, Yang XY, Popescu NC (2010). Synergistic antineoplastic effect of DLC1 tumor suppressor protein and histone deacetylase inhibitor, suberoylanilide hydroxamic acid (SAHA), on prostate and liver cancer cells: perspectives for therapeutics. Int J Oncol.

[160] Chung GE, Yoon JH, Lee JH, Kim HY, Myung SJ, Yu SJ, Lee SH, Lee SM, Kim YJ, Lee HS (2011). Ursodeoxycholic acid-induced inhibition of DLC1 protein degradation leads to suppression of hepatocellular carcinoma cell growth. Oncol Rep.

[161] Murphy DJ, Junttila MR, Pouyet L, Karnezis A, Shchors K, Bui DA, Brown-Swigart L, Johnson L, Evan GI (2008). Distinct thresholds govern Myc’s biological output in vivo. Cancer Cell.

[162] Larsson LG, Henricksson MA (2010). The Yin and Yang functions of the Myc oncoprotein in cancer development and as targets for therapy. Exp Cell Res.

[163] Lin CP, Liu CR, Lee CN, Chan TS, Liu HE (2010). Targeting c-Myc as a novel approach for hepatocellular carcinoma. World J Hepatol.

[164] Brooks TA, Hurley LH (2010). Targeting MYC expression through G-quardruplexes. Genes Cancer.

[165] Llovet JM, Ricci S, Mazzaferro V, Hilgard P, Gane E, Blanc JF, de Oliveira AC, Santoro A, Raoul JL, Forner A, Schwartz M, Porta C, Zeuzem S, Bolondi L, Greten TF, Galle PR, Seitz JF, Borbath I, Haussinger D, Giannaris T, Shan M, Moscovici M, Voliotis D, Bruix J (2008). SHARP Investigators Study Group: Sorafenib in advanced hepatocellular carcinoma. N Engl J Med.

[166] Llovet J M, Bruix J (2008). Molecular targeted therapies in hepatocellular carcinoma. Hepatology.

[167] Cao Z, Fan-Minogue H, Bellovin DI, Yevtodiyenko A, Arzeno J, Yang Q, Gambhir SS, Felsher DW (2011). MYC phosphorylation, activation, and tumorigenic potential in hepatocellular carcinoma are regulated by HMG-CoA reductase. Cance Res.

[168] Kawata S, Yamasaki E, Nagase T, Inui Y, Ito N, Matsuda Y, Inada M, Tamura S, Noda S, Imai Y, Matsuzawa Y (2001). Effect of pravastatin on survival in patients with advanced hepatocellular carcinoma. A randomized controlled trial. Br J Cancer.

[169] Homma Y, Emori Y (1995). A dual functional signal mediator showing RhoGAP and phospholipase C-delta stimulating activities. EMBO J.

[170] Ponting CP, Aravind L (1999). START: a lipid-binding domain in StAR, HD-ZIP and signalling proteins. Trends Biochem Sci.

[171] Wang H, Han H, Mousses S, Von Hoff DD (2006). Targeting loss-of-function mutations in tumor-suppressor genes as a strategy for development of cancer therapeutic agents. Semin Oncol.

